# Novel Coronavirus Pneumonia Treatment With Traditional Chinese Medicine: Response Philosophy in Another Culture

**DOI:** 10.3389/fpubh.2020.00385

**Published:** 2020-07-10

**Authors:** Zhuman Li, Chuangchuang Han, Huihong Huang, Zhijun Guo, Feng Xu

**Affiliations:** ^1^Fengxian Hospital and School of Pharmaceutical Sciences, Southern Medical University, Shanghai, China; ^2^Fengxian Hospital and School of Medicine, Anhui University of Science and Technology, Shanghai, China; ^3^Fengxian Hospital of Traditional Chinese Medicine, Shanghai, China

**Keywords:** COVID-19, pandemic, response, traditional Chinese medicine, modern medicine, cultural difference

In late 2019, novel coronavirus (SARS-CoV-2) caused pneumonia in Wuhan was spread to the whole country and was identified by World Health Organization (WHO) as “public health emergencies of international concern” (1–4). On the morning of March 12, 2020 Beijing time, WHO officially identified it as a pandemic^1^. Up to June 15, 2020, the novel coronavirus disease (COVID-19) has swept over 200 countries and territories, resulting in more than 7.6 million confirmed cases and over 0.42 million confirmed deaths^2^. The novel coronavirus-caused pneumonia has a powerful infectious force for some population groups, and up to now no specific drugs could cure it (5, 6).

Since the novel pneumonia outbreak, China National Health Committee issued seven editions of diagnosis and medical treatment plan^3^. More than 40,000 medical staffs including traditional Chinese medicine (TCM) doctors from all over the country were called up to Wuhan, and other cities in Hubei provinces to treat patients^4^. The epidemic situation displays a good trend after severe prevention and control in China^5^.

In the 7th edition of diagnosis and medical treatment plan issued by National Health Commission (NHC) of China, many TCM remedies are recommended for COVID-19 patients in medical observation period. Huo Xiang Zheng Qi capsule is recommended for patients when there is clinical manifestation of “fatigue accompanied by gastrointestinal discomfort”; and Jin Hua Qing Gan granule, Lian Hua Qing Wen capsule, and Shu Feng Jie Du capsule are recommended for patients when fatigue with fever occur. According to a news release from the National Administration of TCM, the integration of traditional Chinese and Western medical treatment can achieve satisfactory results for resolution of symptoms of COVID-19^6^.

In the medicine field of China, there is always a dispute between the modern medicine and the traditional medicine for a long time. The pros and cons of debate have its own perspective and opinion. We are pleased to see that in the face of severe epidemic situation, there are mixed teams of modern medicine doctors and TCM doctors. The majority of COVID-19 patients in China have been treated with integrated Chinese and modern medicine. Hundreds of herbal remedies have been used throughout the country (7). The Chinese government and academic experts in herbal medicine have recommended incorporating TCM into conventional treatment methods so as to generate synergistic effect by the combinational therapy of Chinese and Western medicine (7).

Unfortunately, the experience of TCM in the treatment of epidemic situation has not been widely recognized and used for reference in the western Occident developed countries. The lack of high-quality scientific evidence may be one of important reason that would lead to reject. Another fundamental reason is that the whole theory system of TCM is not acknowledged by western–trained audience. It might be due to different culture, more specifically, different treatment philosophy. TCM has its strong material base from single monomeric compounds to Chinese herb extracts in COVID-19 treatment. Psychosocial pharmacological effect probably plays an important role in the traditional medicine (8).

So what is the exactly treatment philosophy in TCM culture to the novel coronavirus disease?

Chinese public in general are always long taught that TCM is a national quintessence with an ancient historical origin. In addition to TCM, Peking Opera, martial arts, and calligraphy are well-known as the “four quintessence of China” both at home and abroad. National quintessence itself is more related with culture and social custom than with natural science. TCM has been played an indispensable role in the prevention and treatment of epidemic diseases in history. During the SARS epidemic in 2003, the intervention of TCM has also achieved therapeutic effect (9, 10). In the broad and profound TCM theory system, the present COVID-19 is just one of common epidemics. Even COVID-19 is brand new emerging severe infectious disease caused by a brand new coronavirus and no specific drug is used to cure in modern medicine, TCM still has confidence to fight the epidemic.

In TCM culture perspective, COVID-19 is an epidemic disease caused by an epidemic evil with dampness and heat, which is called Li-Qi in Chinese (11). After Li-Qi invades the human body, it enters the lung first to make the Lung-Qi (vital essence of lung, which is in charge of breath function) stagnate, then lead abnormal breath movement, phlegm-heat accumulation and block, and finally bring out the dead Yin and the dead Yang (12). According to TCM treatment philosophy, dampness should be eliminated first, and then heat be cleared away. After heat and phlegm are cleared away, the body is restored to normal function at last (13).

There seems to be something in common between virus and Li-Qi. Both think that there is an external cause of disease. Modern medicine refer it virus, they usually hope to find specific drug to cure the disease. However, TCM does not know microbiology and could not capture the virus entity, they could only focus on Li-Qi-induced symptom with herbal remedies. The aim of TCM treatment is simple, so long as TCM remedies provide effective way to regulate functional disorders of the human body. Therefore, TCM remedies are used to detoxify poisonous dampness and heat, to strengthen body to resist pathogenic factor, to adjust the harmony of the internal relationship of the human body. That is to say, when TCM doctors treat this kind of disease, they do not have to make the cause clear to start. It is of importance to solve the symptoms for most patients.

A small, non-randomized, single center retrospective observational study reported a shorter average duration of viral shedding and faster resolution of radiological pneumonia in hospitalized COVID-19 patients prescribed Jin Hua Qing Gan granules for more than 2 days as compared with those receiving conventional care (14). The potential efficacy of this herbal medicine for COVID-19 treatment should be further investigated in adequately powered randomized controlled trials.

The understanding and description of TCM is based on the ancient macro understanding of nature and the use of speculative philosophy such as Yin-Yang. In the course of history, since TCM started and developed without synchronizing with modern chemistry, biology and physics, it had to takes the road of philosophical thinking. However, by the aid of advanced science, modern medicine embarks another road of development. It can be said that TCM and modern medicine are two trees growing up in the soil of two different cultures.

Although high-quality clinical trial evidence is lacking at present, the efficacy of TCM remedy on symptom improvement cannot be ignored. To treat COVID-19, TCM and modern medicine should complement each other and cooperate with each other since TCM can contribute as an alternative measure.

## Author Contributions

ZL, CH, HH, ZG, and FX were responsible for the design of this work and interpretation of finding and drafting the work. FX approved the final version to be published and is accountable for all aspects of the work to ensure its accuracy and integrity. All authors contributed to the article and approved the submitted version.

## Conflict of Interest

The authors declare that the research was conducted in the absence of any commercial or financial relationships that could be construed as a potential conflict of interest.

## Footnotes

^1^https://www.sohu.com/a/379386303_162522 (accessed May 15, 2020).

^2^https://www.who.int/emergencies/diseases/novel-coronavirus-2019 (accessed June 15, 2020).

^3^http://www.nhc.gov.cn/xcs/zhengcwj/202003/46c9294a7dfe4cef80dc7f5912eb1989.shtml (accessed June 15, 2020).

^4^http://www.nhc.gov.cn/xcs/fkdt/202004/a116aa8a88034744a001d5d2b06f6fdf.shtml (accessed June 15, 2020).

^5^http://www.nhc.gov.cn/xcs/yqtb/202006/cbbf99e3237645bc87c0dd3b8e99f01b.shtml (accessed June 15, 2020).

^6^http://www.satcm.gov.cn/xinxifabu/meitibaodao/2020-05-13/15078.html (accessed June 15, 2020).
